# Ataxin-1 Fusion Partners Alter PolyQ Lethality and Aggregation

**DOI:** 10.1371/journal.pone.0001014

**Published:** 2007-10-10

**Authors:** Tina Rich, Archana Varadaraj

**Affiliations:** Department of Pathology, University of Cambridge, Cambridge, United Kingdom; National Institutes of Health, United States of America

## Abstract

Intranuclear inclusion bodies (IBs) are the histopathologic markers of multiple protein folding diseases. IB formation has been extensively studied using fluorescent fusion products of pathogenic polyglutamine (polyQ) expressing proteins. These studies have been informative in determining the cellular targets of expanded polyQ protein as well as the methods by which cells rid themselves of IBs. The experimental thrust has been to intervene in the process of polyQ aggregation in an attempt to alleviate cytotoxicity. However new data argues against the notion that polyQ aggregation and cytotoxicity are inextricably linked processes. We reasoned that changing the protein context of a disease causing polyQ protein could accelerate its precipitation as an IB, potentially reducing its cytotoxicity. Our experimental strategy simply exploited the fact that conjoined proteins influence each others folding and aggregation properties. We fused a full-length pathogenic ataxin-1 construct to fluorescent tags (GFP and DsRed1-E5) that exist at different oligomeric states. The spectral properties of the DsRed1-E5-ataxin-1 transfectants had the additional advantage of allowing us to correlate fluorochrome maturation with cytotoxicity. Each fusion protein expressed a distinct cytotoxicity and IB morphology. Flow cytometric analyses of transfectants expressing the greatest fluorescent signals revealed that the DsRed1-E5-ataxin-1 fusion was more toxic than GFP fused ataxin-1 (31.8±4.5% cell death versus 12.85±3%), although co-transfection with the GFP fusion inhibited maturation of the DsRed1-E5 fluorochrome and diminished the toxicity of the DsRed1-E5-ataxin-1 fusion. These data show that polyQ driven aggregation can be influenced by fusion partners to generate species with different toxic properties and provide new opportunities to study IB aggregation, maturation and lethality.

## Introduction

Aberrant protein folds, expressed in post mitotic cells such as neurons, are the precursors of multiple neurodegenerative diseases [1]. A characteristic of the proteins that express these pathogenic folds, for example polyglutamine (polyQ) containing proteins, is their aggregation into intra-nuclear inclusion bodies (IBs). However the role played by IBs in the disease process remains controversial [2] and IBs may be preceded by toxic oligomers [3], [4]. The expression of pathogenic ataxin-1 protein carrying expanded tracts of polyQ results in Spinocerebellar Ataxia type 1, a disease that decimates cerebellar Purkinje cells and brain stem neurons [5]. The existence of heterogeneous IBs nucleated by expanded ataxin-1 suggests multiple aggregation pathways or oligomeric precursors and the possibility to influence ataxin-1 folding. We now investigate this possibility using fusion proteins of ataxin-1.

We chose to study the toxic species of ataxin-1 that expresses an expanded polyQ repeat as our aim was to modulate the toxicity of this species using protein domains distal to the repeat. Our aim was not to compare the toxicity of ataxin-1 proteins that express different polyQ repeat lengths as these studies have been completed by numerous groups and would not be informative for this study of pathogenic ataxin-1 fusions. Instead we chose to exploit the finding that linked proteins alter each others folding pathways [6]–[8]. A protein that aggregates will retard the folding of Green Fluorescent Protein (GFP) [9]. Likewise the substitution of GFP with another tag that exists as a higher order oligomer could alter the folding of a pathogenic protein to which it is fused, and potentially its lethality. Thus far the research impetus has been to increase the solubility of polyQ proteins in an effort to decrease toxicity, as IB formation is regarded by many to be a cytotoxic event. However, oligomeric species of polyQ may be the bona-fida toxic species and much recent data would support this notion [3], [6], [8]. If this is the case, accelerated aggregation, depleting oligomeric ataxin-1 species, may be cytoprotective. We explored this possibility by expressing cDNAs of *A. victoria* EGFP and *Discosoma* DsRed1-E5 fused upstream of an ataxin-1 construct that encodes eighty-two contiguous polyglutamines. GFP naturally exists as a monomer/dimer whilst DsRed1-E5 is an obligate tetramer/octamer [10], [11]. Having engineered and expressed fusion proteins of both GFP and DsRed1-E5 fused at their carboxyl termini to ataxin-1 expressing an expanded polyQ repeat we found that transfectants of either species expressed conspicuous intra-nuclear IBs, though the fluorescent nucleoplasmic fraction typically seen in GFP-ataxin-1 transfectants was absent in DsRed1-E5-ataxin-1 transfectants. The kinetics of IB deposition was modified by the different fusion species, though both species of IB could sequester PML-NDs, a finding which suggests a common pathogenic property of these and perhaps many other proteinaceous aggregates. Transfectants expressing DsRed1-E5-ataxin-1 suffer reduced viability compared to those expressing GFP-ataxin-1; which could be reversed by co-expression with GFP-ataxin-1 or expression of a shortened glutamine repeat. Collectively, these data show that the biologic properties of IBs seeded by the same pathogenic ataxin-1 protein can be markedly altered by its fusion to different protein tags and that accelerated IB formation was not cyto-protective in this experimental system.

## Results

### Altered expression pattern and IB morphology of ataxin-1 fusion proteins

Untagged, GFP and DsRed1-E5 fusions of a pathogenic ataxin-1 construct were transiently transfected into HeLa cells. DsRed1-E5 is a mutant of the DsRed1 protein and fluoresces green when first synthesised, then after a few hours, red [11]. This colour change is contingent on DsRed1-E5 folding into its tetramer/octameric state [12]. Fluorescence microscopy of ataxin-1 transfectants, with immunostains for untagged ataxin, revealed striking variations in expression pattern for the fusion proteins. Unless otherwise stated, ataxin-1 refers to ataxin-1 expressing an expanded glutamine repeat with 82 glutamines.

**Figure 1:**
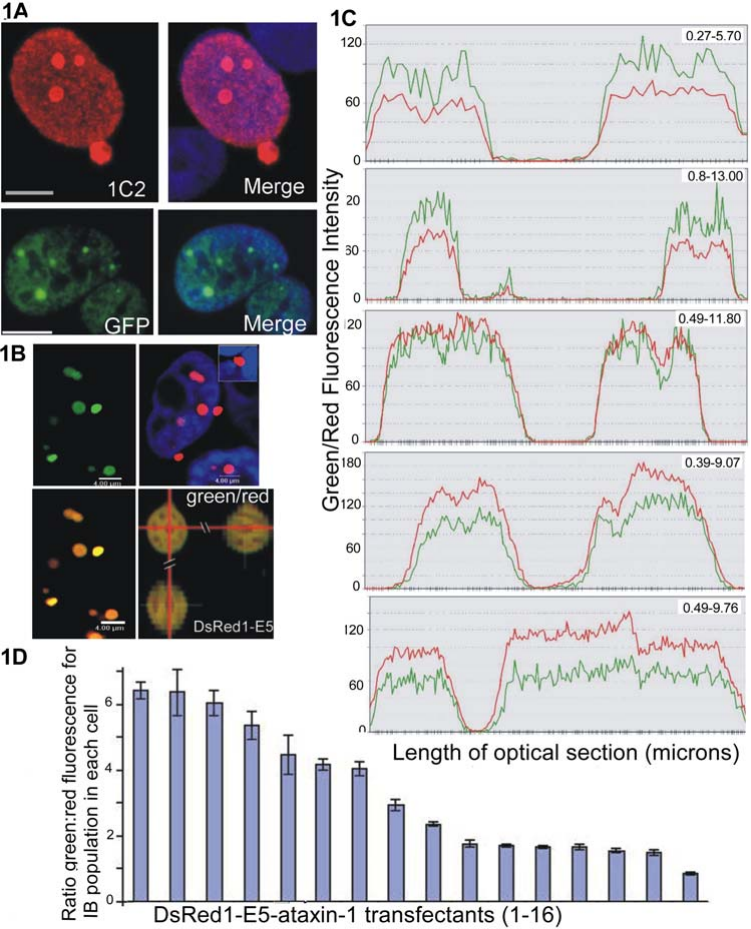
Expression patterns of ataxin-1 fusion proteins. Figure 1A. Distribution of ataxin-1 and ataxin-1-GFP. Upper panel; untagged ataxin-1 stained with 1C2 with a DAPI merge; lower panel, GFP-ataxin-1 with DAPI merge. Figure 1B. DsRed1-E5-ataxin-1 emits both red (r) and green (g) fluorescence. The merged red green and red/DAPI fluorescence micrographs are also shown. An orthogonal slice through a reticular IB seeded by DsRed1-E5-ataxin is shown lower right. Scale bars: 8 microns. Figure 1C. IBs of DsRed1-E5-ataxin express protein of comparable age. Synchronous peaks and troughs of red/green fluorescence emitted by IBs of DsRed1-E5-ataxin in shared nuclei. IBs are ranked according to maturity; with high g/r ratios indicating immature protein. Figure 1D. Synchronous seeding of DsRed1-E5-ataxin IBs. HeLa nuclei were ranked according to mean g/r ratio of their IB populations (24hr after transfection). 169 IBs in 16 nuclei were included in this data set.

Untagged ataxin-1 (immuno-stained with an anti polyQ monoclonal antibody 1C2) and GFP fused ataxin-1 were found to be expressed as both nuclear IBs and dispersed throughout the nucleoplasmic fraction as granular material that excluded the nucleoli (Figure 1A). Nucleoplasmic ataxin-1 is thought to comprise oligomeric species that precede IB formation; a notion supported by the expression of nucleoplasmic GFP-ataxin-1 in nuclei entirely devoid of IBs and its diminution as IBs nucleate [10]. In GFP-ataxin-1 transfectants, the majority of cells (82%±3 (n = 100)) expressed both nucleoplasmic and IB nucleated ataxin-1 with the remaining cells expressing a nucleoplasmic fraction only. 75±8% (n = 100) of GFP-ataxin-1 containing IBs were dense with no obvious substructure. The remainder were reticular as we have previously shown [13]. Interestingly, IB morphology was nucleus specific, suggesting a shared driver such as local protein concentration. Note that for ataxin-1, expression of an expanded glutamine repeat in cell culture increases the frequency of nuclei that express large IBs (≥ 1 micron in diameter). The majority of transfectants express IBs whether transfected with normal or expanded ataxin-1 [14], with the frequency of large IBs diminished in transfectants expressing ataxin-1[Q30].

In contrast to the GFP fusion, DsRed1-E5-ataxin-1 was expressed as reticular intranuclear and cytoplasmic IBs similar to those reported for nuclear aggresomes [15], aggregates of RED protein [13] and certain cytoplasmic puncta (Figure 1B) [16]. High magnification imaging at low voltage revealed the reticular IB structure which can be obscured by pixel saturation at higher voltages (Figure 1B, lower right). The lack of a diffuse or oligomeric nucleoplasmic fraction, as suggested by the loss of fluorescence in this compartment, was confirmed by immunostains with the 1C2 antibody (data not shown). As for the GFP fusion, transfectants expressing DsRed1-E5 fused to ataxin-1 expressing a normal polyQ repeat length [Q30] expressed smaller IBs, still with no evident nucleoplasmic fraction (data not shown). Both green and red fluorescence, attributable to immature and mature DsRed1-E5, was evident in each IB, regardless of size. Large intact cytoplasmic IBs of DsRed1-E5-ataxin-1 frequently deformed the nuclear envelope (Figure 1B, inset), in common with nuclear pore complexes seen in Alzheimers disease [17]. The perinuclear location of these proteins prompted us to address whether they reorganised vimentin–a characteristic of the aggresome. However, immunostains for vimentin failed to indicate its disruption by cytoplasmic IBs of DsRed1-E5-ataxin-1 (data not shown).

### DsRed1-E5-ataxin-1 IBs nucleate synchronously

**Figure 2:**
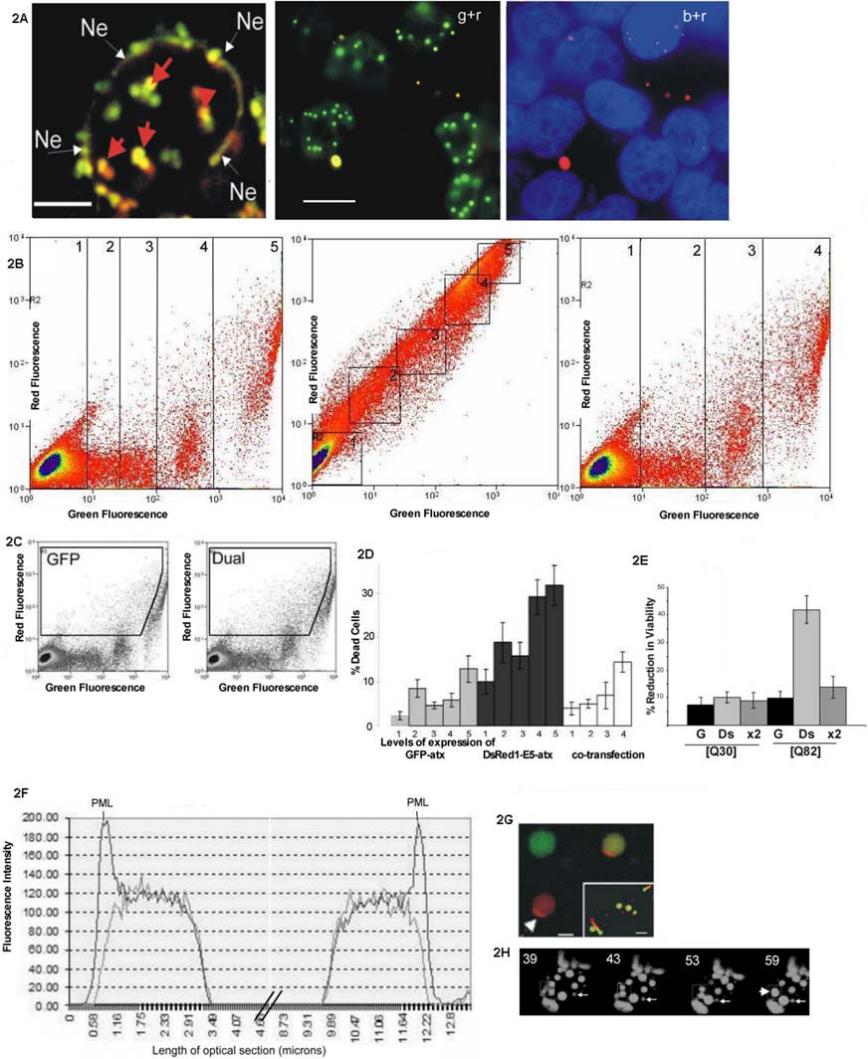
IB properties and lethality of ataxin-1 fusion proteins. Figure 2A. Distribution of DsRed1-E5-PML and dual transfected ataxin-1 fusions. LH panel; puncta of DsRed1-E5-PML reveal heterogeneous red/green fluorescence (arrowed) in a HeLa transfectant. Ne denotes nuclear envelope. Scale bar: 8 microns. RH panel; cytoplasmic enrichment of aged DsRed1-E5-ataxin in dual transfectants. Green and red indicates the wavelengths scanned in each micrograph, with DAPI co-stain. Scale bar: 15 microns. Figure 2B. Red/green fluorescence in single and co-transfectants of ataxin-1 fusion proteins. LH panel; GFP-ataxin-1; Middle panel; DsRed1-E5-ataxin-1; RH panel; dual transfectants. Figure 2C. Enriched red fluorescence in co-transfectants occurs in the region indicated. Figure 2D. Dye uptake shows increased lethality of DsRed1-E5-ataxin-1. Increased DAPI uptake is seen in DsRed1-E5-ataxin-1 versus GFP-ataxin-1 and Dual transfectants. Figure 2E. Cell proliferation assay shows reduced viability in DsRed1-E5-ataxin-1 transfectants. MTT based assay of DsRed1-E5 (Ds), GFP (G) and dual (x2) transfectants, comparing ataxin-1 expressing both normal [Q30] and expanded polyQ repeats [Q82]. Figure 2F. PML-NDs are sequestered by IBs of DsRed1-E5-ataxin-1. Histogram of red/green fluorescence across a DsRed1-E5-ataxin-1 transfectant, stained with N19. Spikes of red fluorescence (darker line) denote PML-NDs tethered to IBs in a single nucleus. Figure 2G. PML/DsRed1-E5-ataxin-1 sequestration. Scale bar: 8 microns. Inset; ataxin-1/PML sequestration captured by immunofluorescent staining of endogenous PML (red) with GFP-ataxin-1. Scale bar: 15 microns. Figure 2H. Mobility of DsRed1-E5-ataxin-1 IBs revealed by time-lapsed confocal microscopy. 9-section Z series were collected every 5 minutes for an hour. The four frames shown indicate fusion events over twenty minutes in a single optical section. Multiple fusion events occur within the boxed region. The arrowed IB also tracks towards this region. The disordered fluorescence at the extremities of the nucleus corresponds to cytoplasmic material.

The existence of heterogeneous IBs pointed to a role for kinetics in shaping inclusion body morphology and ataxin-1 distribution. To address this we took advantage of the spectral properties of DsRed1-E5 to investigate the seeding process itself. DsRed1-E5′s colour transition is independent of protein concentration, allowing us to discriminate between newly synthesized versus mature protein. This property would also allow us to study the mode of aggregation, for example by identifying a core of older protein. Confocal microscopy was used to measure the fluorescence intensity across equatorial sections (at .05/.06 micron intervals) of individual IBs in DsRed1-E5-ataxin-1 transfectants (Figure 1C). Ratios of green and red fluorescence were found to be highly conserved across individual inclusions (Figure 1C, Table 1) with synchronous spikes and troughs detected at both wavelengths. A striking intranuclear but not internuclear conservation of g/r ratio was also apparent (Figures 1C and 1D, Table 1). However there was no clear relationship between IB diameter and g/r ratio (Table 2). These data suggested the synchronous deposition of IB populations in each nucleus. To test the influence of the DsRed1-E5 moiety as a driver for synchronous IB seeding we expressed a second DsRed1-E5 fusion, this time to the promyelocytic leukaemia protein (PML) [18]. PML is the principle structural constituent of PML nuclear domains, stress responsive polymeric structures that are disrupted by protein aggregates [19], [20]. In contrast to DsRed1-E5-ataxin-1, fluorescence microscopy of nuclear puncta seeded by DsRed1-E5 fused to PML revealed bodies of quite different maturity and fluorescence (Figure 2A). Clearly then, fusion to DsRed1-E5 alone is not sufficient to result in simultaneously seeded structures.

**Table 1:** Green/Red Fluorescence ratios in IBs of DsRed1-E5-ataxin-1 IBs.

IB Diameter (µm)		g/r Fluorescent Ratio (mean±SD)
Nucleus 1	1.98	1.56±0.29
	2.32	1.54±0.24
Nucleus 2	2.09	0.56±0.08
	2.38	0.63±0.11
Nucleus 3	4.33	0.87±0.10
	3.20	0.94±0.13

Equatorial line scans through IBs were used to generate g/r ratios. Mean g/r fluorescence ratios are shown for three pairs of IBs (in separate nuclei). Fluorescence intensities were measured at .05/.06 micron intervals. SD: standard deviation.

**Table 2:** Non-linear relationship between g/r ratio and IB diameter.

IB Diameter (µm)	g/r Fluorescence Ratio (mean±SD)
1.1–1.9	6.39±0.74
0.9–1.8	6.34±1.35
1.3–1.5	6.03±1.13
1.2–2.0	5.34±0.93
1.0–1.9	4.47±1.56
1.2–2.3	4.16±0.53
1.3–2.3	4.03±0.66
1.1–2.6	2.91±0.48
2.0–2.6	2.33±0.16
0.8–2.8	1.74±0.28
1.2–3.0	1.68±0.10
1.4–3.5	1.65±0.11
1.6–4.1	1.64±0.18
1.6–4.1	1.53±0.11
1.4–2.8	1.48±0.14
3.0–3.8	0.85±0.10

Mean fluorescence ratios calculated for the IBs populations shown in Figure 1C. For each nucleus the mean g/r ratio is shown, with the diameter of the largest and smallest IB in that nucleus. These data reveal the lack of any linear relationship between g/r ratio and IB size. SD: standard deviation.

### Maturation of the DsRed1-E5 fluorochrome is inhibited by co-expression with GFP-ataxin-1

Expression of ataxin-1 was also examined in co-transfected Hela nuclei to assess the dominance of either fusion in altering viability. Twenty-four hours after co-transfection into HeLa cells the predominant expression pattern (in 78±4%) were cells expressing intense diffuse green nucleoplasmic fluorescence and intranuclear IB's with emission maxima characteristic of GFP-ataxin-1. Occasional red fluorescence was as cytoplasmic IBs or small punctate intranuclear aggregates (Figure 2A). Cytoplasmic IBs of mature DsRed1-E5-ataxin-1 are found in single transfectants (Figure 1A) and arise as protein formed during the first interphase after transfection becomes trapped in the cytoplasm after mitosis (confirmed by cell cycle synchronisation experiments-data not shown). However, the striking and reproducible difference between single and double transfectants expressing DsRed1-E5 fused ataxin-1 was the reduction of intranuclear red fluorescence. These data suggested a model in which maturation of DsRed1-E5-ataxin-1 was inhibited in the nuclear compartment, when co-expressed with the GFP fused ataxin-1. To quantitatively test this we exploited the difference in emission spectra between GFP and DsRed1-E5 to identify the dominant fluorescent signature in double transfectants. Flow cytometric density plots of GFP-ataxin-1 transfectants (Figure 2B) revealed green fluorescent cells of low to high fluorescence intensity (denoted by boxed regions 1–5). The red fluorescence in transfectants with the highest green fluorescent intensity is due to the compensation level, which could not be increased if the less fluorescent cells were not to dip beneath the detection threshold. The degree of compensation used was sufficient to allow all fluorescent events to be visualised. The clustering of fluorescent events in boxes 4 and 5 (a consistent event) may indicate the fluorescence due to folding intermediates.

DsRed1-E5-ataxin-1 transfectants revealed cells arrayed on a diagonal (Figure 2A, middle panel), with immature green fluorescent DsRed1-E5-ataxin-1 becoming redder (and greener) with increasing time after transfection. Again these cells could be grouped according to increasing fluorescence intensity and each group could be analysed individually for viability. In double transfectants (Figure 2B, RH Panel) the fluorescence signature attributable to DsRed1-E5-ataxin-1 is ablated, and a dominant green fluorescent signature, characteristic of GFP-ataxin-1 evident (Figure 2B, right hand panel). Only a small increase in cell number is seen in the region indicated in the two density plots (Figure 2C).

### GFP-ataxin-1 expression is cytoprotective for DsRed1-E5-ataxin-1 transfectants

Recent data suggests that oligomeric forms of polyQ protein are cytotoxic [3], [6], [21]. The preference for IB formation by the DsRed1-E5 versus GFP fused ataxin-1 protein suggested to us that the DsRed1-E5 fusion aggregated differently. To assess whether enhanced IB formation also impacted cytotoxicity we tested cell viability using two assays. The first is an MTT based assay that measures cellular reducing power as a correlate of viable cell number. The second assay measures dye uptake, with reduced viability leading to increased membrane permeability and therefore dye positive cells. These methodologies were chosen as they do not bias towards any particular form of cell death; being inclusive of both secondary apoptotic and necrotic events. Viability was also correlated with the ablation of fluorochrome maturation in double transfectants. Cells with high versus low fluorescence intensity for either fusion protein were selected (boxed regions in Figure 2B) and their DAPI uptake assessed to determine membrane integrity. Cells expressing the highest fluorescent signal attributable to GFP-ataxin-1 (box 5) were found to have a dye permeable fraction of 12.85±3% (Figure 2D). Similar measurements with DsRed1-E5-ataxin-1 transfectants showed an increased dye uptake of 31.8±4.5% of cells (Figure 2D). In double transfectants viability was comparable to the GFP-ataxin-1 expressing cells (dye permeable cells; 14.4±2.3%). These trends were comparable with a second viability assay that measures the reducing power of identical numbers of each transfectant pool (Figure 2E). However, equivalent viability assays for GFP and DsRed1-E5 fused ataxin-1[Q30] revealed low levels of toxicity for either fusion species. In the case of normal polyQ repeat lengths, the DsRed1-E5 moiety had no significant effect on toxicity.

### PML-NDs are sequestered by both species of ataxin-1 fusion proteins

One property of pathogenic ataxin-1 protein is to sequester and disrupt specific nucleoproteins such as promyelocytic leukaemia nuclear domains (PML-NDs) [18], [20], [22]. We tested whether sequestration of PML-NDs was demonstrable for IBs seeded by DsRed1-E5-ataxin-1, as previously shown for GFP-ataxin-1. Using DsRed1-E5-ataxin-1′s property of emitting dual red and green fluorescence combined with immunostains with a pan PML antibody we identified sequestered PML-NDs (Figures 2F/G). Sequestered PML is evident in the fluorescent micrograph in Figure 2F. 92±5% of IBs over 1 micron in size sequestered PML-NDs. Previously we showed irreversible PML-ND sequestration by protein inclusions in stressed cells [20]. Similar assays for the irreversibility of PML-ND sequestration following heat shock revealed sequestered PML-NDs on 90±6% of DsRed1-E5-ataxin-1 IBs and 93±4% of GFP-ataxin-1 IBs of over 1 µm in diameter.

## Discussion

IBs are the terminal stage of a series of aggregation steps, leading us to predict that IB morphology, lethality, and formation kinetics may reflect the preceding aggregation processes. We now demonstrate this biologic heterogeneity using IBs seeded by an identical polyQ repeat expressed in the context of different fluorescent protein tags.

It is widely thought that the toxicity of polyQ proteins is, in part, due to their sequestration of nucleoproteins such as PML-NDs and typical PML-NDs are found in several neuronal species [23], [24]. Irreversible sequestration of nucleoprotein effectors increases cellular sensitivity to injury [25]. For both ataxin-1 fusions we identified irreversibly bound PML-NDs following heat shock, a stress that ordinarily fragments PML-NDs [20]. PolyQ diseases are thought to be a disease of transcription. Crucially, PML-NDs contain cargo proteins, amongst them chromatin modifiers, that are released into the nucleoplasm following PML-ND fragmentation [19]. Disruption of these processes may be cytotoxic to the cell and, in the context of our experiments, may be provoked by either ataxin-1 fusion expressing an expanded repeat. The question arises as to why PML-NDs are so susceptible to disruption by protein aggregates. One possibility is that PML-NDs are part of the intranuclear apparatus to monitor abnormal accumulations of proteins [26] and nucleic acid, in a similar fashion to cytoplasmic Trim5 [27]. As such, PML-NDs may be recruited to sites of protein deposition, which results in their sequestration. The recruitment of PML-NDs to sites of ionising radiation induced foci (IRIF) may be another example of PML-NDs detecting high concentrations of protein and disturbed chromatin [28]. Recent data has shown the translocation of PML towards sites of viral intrusion [29]. The inset micrograph in Figure 2G may indicate a similar translocation of PML protein, though this would have to be confirmed by live cell imaging which is currently unreliable given the artefactual activity recorded for ectopic PML transfectants [28].

Co-expression of both ataxin-1 fusions resulted in a dramatic loss of red fluorescence, probably through sequestration of immature DsRed1-E5-ataxin-1 by GFP-ataxin-1, which may then be locked into an unfavourable fold for maturation. This may explain the small diameter of red puncta in double transfectants; presumably size limited by an emergent pool of GFP-ataxin-1. The loss of red fluorescence does not imply that the DsRed1-E5-ataxin-1 protein has not aggregated. Rather it shows that the folding pathway followed by this fusion form of ataxin-1 is such that the DsRed1-E5 moiety has failed to form a tetramer, which is necessary for it to fluoresce. Critically, it is this failure to express red fluorescence which correlates most strongly with lethality. The co-aggregation of DsRed1-E5-ataxin-1 with GFP-ataxin-1 protein probably prevents DsRed1-E5 tetramer formation. One caveat to these data is that the detection of small fluorescent puncta by flow cytometry is less efficient than that of larger aggregates or diffusely expressed material; which could lead to the under-estimation of DsRed1-E5-ataxin-1 puncta. None-the-less, the loss of the DsRed1-E5 fluorescence signature in double transfectants is a potent illustration of how different species of fusion protein can influence each-others folding characteristics.

Our experimental strategy also allowed us to investigate IB seeding as we could retrospectively gauge the maturity of protein within individual IBs. The fluorescence profiles of DsRed1-E5-ataxin-1 IBs suggested that they synchronously nucleate from material of similar maturity. Mobility and fusion of large intranuclear IBs [16], [30] also supports the notion that IB resident proteins are not wholly insoluble and that IB interaction continues after initial seeding events, so preserving the green/red fluorescence ratios. The mobility of DsRed1-E5-ataxin-1 IBs would support this notion (Figure 2H).

The lethality of the DsRed1-E5-ataxin-1 fusion was evident by dye uptake and metabolic assays, even at low levels of fluorescence, and was attenuated in double transfectants. These data are consistent with the generation of a toxic DsRed1-E5-ataxin-1 species whose toxicity is diminished by co-expression with GFP-ataxin-1. The reduction of toxicity in double transfectants and the minimal maturation of the DsRed1-E5 fluorochrome suggests that the two events are linked. Folding of the DsRed1-E5 fluorochrome and a mature red fluorescent signal was associated with toxicity. At this stage we do not know if the intermediate folding species are the same for either aggregate; future work will address these issues. MTT based assays showed similar toxicities for DsRed1-E5 and GFP fused ataxin-1[Q30]. Recent work [6] has suggested that fusion tags can act as templates for the aggregation of polyQ repeats, with expanded repeats acting to accelerate aggregation. Our data would agree with this hypothesis. Additionally the DsRed1-E5 template only serves as a primer for a toxic folding pathway when expressed with an expanded polyQ repeat. Future work will allow us to generate a bank of primers that are both cytoprotective and deleterious to the health of the cell, in order to delineate the aggregation pathways that can be accessed to diminish toxicity.

The toxicity of the DsRed1-E5 fusion of ataxin-1 is consistent with recent reports of other highly toxic reticulated species of inclusion[31]. Whether or not these species arise in polyQ pathologies will need to be addressed. Further experiments will be required to assess the toxicity of DsRed1-E5 in other cell types. The data that we report here for HeLa transfectants was found to be reproducible in neuroepithelial HEK293 cells, though we have not extended these experiments to untransformed neuronal cells. The possibility of altered forms of cell death active in neuronal lineages versus other cell types is another important consideration [32]. Further, the potent inhibition of DsRed1-E5 maturation and toxicity by the GFP fusion form of ataxin-1 may indicate the success with which polyQ species can influence and ultimately disrupt multiple sub-nuclear targets. These in-vitro cell systems are powerful tools with which to test protein aggregation but we must take into consideration the kinetics of protein accumulation in these models. The possibility remains that rapid IB formation in cell culture is an overtly cytotoxic event that masks more subtle underlying pathologies that arise from the sequestration of bystander proteins by polyQ species. Additionally, PolyQ inclusions grow rapidly once seeded, but the initial seeding event is preceded by a lengthy lag phase during which pre-IB polyQ protein is expressed[33]. During this lag phase, the cell may employ injury pathways to adapt to the presence of polyQ protein. These adaptive injury responses may not be expressed in cell culture models because of the rapidity of IB formation. We are currently testing these hypotheses by using PML-NDs as stress markers in cells expressing low amounts of oligomeric polyQ protein.

## Materials and Methods

### Plasmid constructs

GFP-ataxin-1 encoding 82 and 30 contiguous glutamines were gifts from Professor H. Zoghbi (Baylor, USA) and Professor H. Okazawa (Tokyo Medical and Dental University, Japan). DsRed1-E5-ataxin-1and DsRed1-E5/PML (PML-VI) cDNAs were assembled in pTimer (Invitrogen, Paisley, UK) and expressed in pcDNA3.1 (Invitrogen).

### Transient Transfection, Immuno-cytochemistry and Heat Shock

Transient transfection was with Fugene 6 (Roche Applied Science, Basel, Switzerland) and immuno-staining and heat shock was as described previously [20]. 1 µg of each construct was used for transfections, with co-transfections carried out with 2 µg total DNA. To compare dual and single transfectants, an additional 1 µg control–non-fluorescent empty vector was included in each single transfection reaction. Anti-PML (clone N19, Santa Cruz, CA, USA) was used at a dilution of 1/250 and anti-polyQ (clone IC2, Chemicon Europe, Hampshire, UK) at 1/10,000. IB quantitation was by ocular counts of multiple fields using a Zeiss Axiophot fluorescence microscope. At least 100 nuclei were counted for each data set.

### Flow Cytometry and Viability

Cells were scraped from dishes and washed in calcium/magnesium free PBS to avoid clumping. Analysis was either directly or following the inclusion of DAPI for cell viability assays. Acquisition was with a 3 laser MoFlo cytomation cell sorter and analysis was with CELLQuest (Becton Dickinson) and Summit offline v3.1 software. Live/dead discrimination was by the inclusion of 100 ng/ml DAPI reagent. FL5 (DAPI) versus FSC was used to collate live/dead ratios from a gated set that excludes doublets. At least thirty thousand events were collected for each experiment. To ‘back-of’ the fluorescence of high GFP-ataxin-1 expressers (to facilitate compensation), a neutral density condenser was used. The CellTiter 96® AQ_ueous_ one Solution Cell Proliferation Assay (Promega) is a colorimetric method for determining viable cell number (based on the MTT assay). This assay was performed according to manufacturer's instructions using identical cell numbers (10,000) seeded into 96 well plates. 24 hours after transfection, % loss of viability was calculated by measurements of soluble formazan. Fluorescence microscopy was used to confirm equivalence of transfection efficiency.

### Confocal microscopy

Confocal microscopy was with the DM IRBE Leica Confocal microscope. Z series stacks were assembled as maximum projection images using the Leica Confocal Software version 2.5. A 63×1.32 oil immersion objective was used for high magnification image acquisition. For calculations of the ratios of red/green fluorescence we first ensured that fluorescence intensities were not distorted by differential rates of bleaching. Fluorescence ratios were calculated from the mean relative fluorescence intensities over a section delineated through each IB. ‘Glow Over Under’ mode was used to minimise pixel saturation. Time lapse microscopy was with cells seeded on a cover-slip and inserted into a heated chamber. Fields of newly transfected cells were identified by epifluorescence then imaged as Z stack series every 5 minutes by confocal microscopy. HEPES buffered media and phenol-red free media were used. Serial Z series were collected for 9 optical sections, averaged 3 times over three channels, red (568 nm) green (468 nm) and phase contrast, at a resolution of 512×512 and zoom set at 2.4. An HCX PL APO 63.0×1.20 corrected for UV objective was used.
